# An Inside Job: Applications of Intracellular Single Domain Antibodies

**DOI:** 10.3390/biom10121663

**Published:** 2020-12-12

**Authors:** Eline Soetens, Marlies Ballegeer, Xavier Saelens

**Affiliations:** 1 VIB-UGent Center for Medical Biotechnology, VIB, B-9052 Ghent, Belgium; eline.soetens@vib-ugent.be (E.S.); marlies.ballegeer@vib-ugent.be (M.B.); 2 Department of Biochemistry and Microbiology, Ghent University, B-9000 Ghent, Belgium

**Keywords:** single domain antibody, intrabody, delivery methods, therapy, research tool

## Abstract

Sera of camelid species contain a special kind of antibody that consists only of heavy chains. The variable antigen binding domain of these heavy chain antibodies can be expressed as a separate entity, called a single domain antibody that is characterized by its small size, high solubility and oftentimes exceptional stability. Because of this, most single domain antibodies fold correctly when expressed in the reducing environment of the cytoplasm, and thereby retain their antigen binding specificity. Single domain antibodies can thus be used to target a broad range of intracellular proteins. Such intracellular single domain antibodies are also known as intrabodies, and have proven to be highly useful tools for basic research by allowing visualization, disruption and even targeted degradation of intracellular proteins. Furthermore, intrabodies can be used to uncover prospective new therapeutic targets and have the potential to be applied in therapeutic settings in the future. In this review we provide a brief overview of recent advances in the field of intracellular single domain antibodies, focusing on their use as research tools and potential therapeutic applications. Special attention is given to the available methods that allow delivery of single domain antibodies into cells.

## 1. Introduction

**Figure 1:**
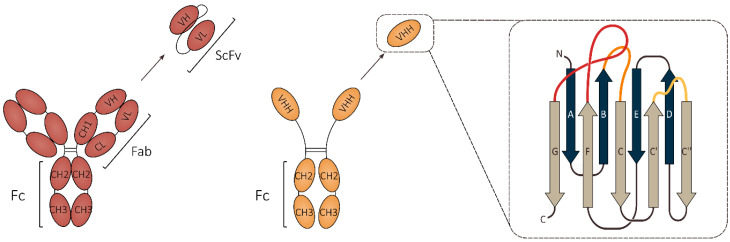
Schematic representation of a conventional IgG antibody (**left**) and a camelid heavy chain antibody (**middle**), with their respective smallest antigen-binding formats: a single chain variable fragment (ScFvs) and a single domain antibody (VHH). To the right, a folded VHH domain is schematically shown with CDR 1, 2 and 3 indicated in orange, yellow and red, respectively. CH: heavy chain constant domain; CL: light chain constant domain; VH: heavy chain variable domain; VL: light chain variable domain; VHH: variable domain of heavy chain antibody.

In 1993, a serendipitous discovery that led to the identification of a new type of antibody in the sera of camelid species was reported [1]. It was demonstrated that the serum of these mammals not only contains classical immunoglobulins (IgGs), but also a second type of antibody that consists only of heavy chains. These so called heavy chain antibodies (HCAbs) make up 50–80% of the total serum IgG amount in camels and 10–25% in South-American camelids [2], suggesting that they play an important role in the humoral immune response of these animals. Similar alternatives to classical antibodies, named new antigen receptor (IgNAR) antibodies, were also found in the sera of some cartilaginous fish, such as nurse sharks and wobbegong sharks [3,4]. Although the sequences of these ancestral IgNAR antibodies differ significantly from camelid HCAbs, their structures show a remarkable resemblance, which points towards convergent evolution [4,5]. In the wake of the discovery of HCAbs in camelid sera, it was soon realized that the HCAb variable domain (VHH) could be expressed as a separate protein fragment, while retaining full antigen binding capacity [6]. VHHs have a size of about 2.5 nm in diameter and 4 nm in length and are the smallest natural antigen-binding entities known to date [7] (Figure 1).

Heavy chain antibodies lack light chains, as well as the first constant domain (CH1). To compensate for these differences, the variable domains of HCAbs have adopted distinct structural features. Firstly, the hallmark residues in framework region 2 (FR2) of the VHH, which account for the interaction with the light chain in classical antibodies, have been replaced by more hydrophilic residues [8,9,10,11]. Indeed, it has been demonstrated that swapping the corresponding hydrophobic residues in a human heavy chain variable domain (VH) for smaller and more hydrophilic ones, promotes solubility and avoids aggregation [12]. The paratope of VHHs consists of three complementarity determining regions (CDRs) compared to six in classical antibodies. The adjacent CDR loops of the heavy and light chain variable domains in classical antibodies form a paratope surface of 600–900 Å^2^. VHHs make up for the reduced number of CDR loops by increasing their length, in particular CDR3 (on average two to four amino acids longer compared to human IgG [9]), resulting in an average paratope surface of 600–800 Å^2^ [13]. As a longer CDR3 loop may be entropically unfavorable for epitope binding, camel-derived VHHs often contain an additional interloop cysteine bridge between CDR1 and CDR3, effectively constraining the movement of the CDR3 loop [14]. This disulfide bond is notably less common in llama VHHs, which can be explained by the shorter CDRs present in these VHHs [9,10,11]. Structural differences between the variable domains of classical antibodies and HCAbs can explain why these two antibody variants differ in their target antigen preference. Classical antibodies typically adopt a concave paratope surface, making them more prone to bind to protruding or flat surfaces. On the other hand, the prolate shape of VHHs combined with their small size makes them very suitable for binding clefts on the protein surface, such as enzymatic sites [13].

VHH coding sequences are usually isolated from immunized animals, after which specific binders are enriched by robust selection methods such as phage display, although other types of VHH libraries (BOX 1) and selection methods (BOX 2) have also been successfully used to identify high affinity binders. Several properties of VHHs have made them attractive research tools and explain why they have been extensively used in many labs in the almost thirty years since their discovery. The classical antibody counterpart of VHHs are called single-chain variable fragments (scFvs) and are made up of the heavy and light chain variable domains of classical immunoglobulins, connected by a flexible peptide linker. Over the years, these scFvs have also proven their value as research tools [15], yet the main advantage VHHs hold over scFvs is that they only consist of one domain, which makes them more convenient for cloning and genetic engineering. Additionally, VHHs with affinities in the nanomolar to picomolar range can be routinely obtained (BOX 2) and can even be improved further by in vitro affinity maturation techniques that are based on error prone PCR or Ala-scanning [16,17]. Lastly, VHHs are usually very stable, being able to resist chemical (e.g., 7 M guanidinium chloride or 10 M urea) and thermal denaturation (T_m_ is on average 60 °C or higher) [18].

## 2. Single Domain Antibodies as Intrabodies

The term intrabodies is derived from *intracellular* and *antibody*, and refers to antibodies that can be used to target selected proteins inside the cell. Originally, intrabodies mainly existed as single-chain variable fragments and were seen as a promising new class of active molecules that could be used for treatment of diseases such as AIDS [19]. Although there are examples where scFvs have been used successfully as intrabodies, the reducing environment of the cytoplasm, in which the intra-domain cysteine bridges fail to form, often prohibits their proper folding [20]. Some proposed solutions for this problem include expression of the scFvs as fusion proteins, e.g., with the *E. coli* maltose binding protein [21], design of scFvs that lack disulfide bridges [22] and grafting of CDRs onto stable scFvs frameworks [23]. Despite these possibilities, the inherent stability of single domain antibodies makes them much more suitable for intracellular expression compared to their scFvs counterparts. Indeed, VHHs in which both cysteine residues were replaced by serine residues were still able to effectively bind their cytosolic targets with only a 2-fold reduction in affinity, as was shown for a VHH targeting glial fibrillary acidic protein whose affinity decreased from 5.6 nM to 12 nM upon mutation of the cysteine residues [24]. It is worth noting that VHHs containing an additional disulfide bridge, which is common in camel VHHs but also occurs in VHHs derived from other species [9,10,11,14], are more likely to fail to fold properly in the cytoplasm and may thus be less suitable for use as intrabodies [25]. Nonetheless, the single domain nature of VHHs makes them decidedly easier to select and engineer, and allows targeting of epitopes that would not be available to scFvs.

Usually, intrabodies are directly expressed inside the cell, yet other delivery methods that facilitate transport of the VHHs across the cell membrane are possible. Once inside the cell, the small size of VHHs allows them to diffuse freely through the nuclear pore complexes, although they can also be specifically targeted towards the nucleus by addition of a nuclear localization signal (NLS) [26]. Furthermore, VHHs can be targeted to the lumen of the endoplasmic reticulum (ER) by fusion with a secretory signal peptide combined with the KDEL ER-retention sequence [27,28]. In this way, VHHs are retained in the ER compartment together with their antigen. Using this method for targeting of secreted or membrane proteins allows modulation of the secretome of the cell, from the inside out.

The propensity of VHHs to bind cleft-like structures makes them ideal for the binding of active sites of enzymes. Intracellular expression of these VHHs opens up a whole new range of potential therapeutic targets, which were previously inaccessible using traditional monoclonal antibody (mAb) therapy. Moreover, intracellular VHHs are also very well suited to study protein function, because their target specificity can be combined with coupling of the VHH to a functional group, such as a proteasomal degradation tag, which allows selective degradation of the target protein. In the next section the advantages and disadvantages of available methods for delivery of VHHs into the cell interior are discussed.

## 3. Delivery Methods for Intracellular Single Domain Antibodies

**Figure 2:**
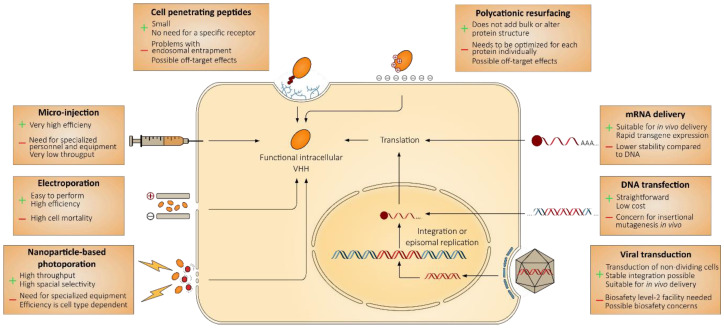
Schematic diagram of methods that are available to deliver VHHs into the cytosol of the cell. Methods such as micro-injection, electroporation and photoporation (**left**) require (transient) physical disruption of the cell membrane, while coupling to cell penetrating peptides and polycationic resurfacing (**top**) are based on electrostatic interactions with the cell surface. Expression based methods include mRNA, DNA or viral vector-based delivery processes (**right**).

The plasma membrane is a barrier that prevents the diffusion of intracellular macromolecules into the cell surrounding medium and, vice versa, the spontaneous uptake of exogenous macromolecules. In order to deliver macromolecules, such as VHHs, to the cytoplasm of the cell, the plasma membrane thus needs to be transiently breached. There are two main methods to achieve this: extracellular addition of purified proteins, often combined with a physical plasma membrane disturbing procedure, or delivery of a genetic element into the target cell cytoplasm or nucleus, which instructs for the intracellular expression of the VHH (Figure 2).

### 3.1. Microinjection, an Efficient but Low-Troughput Method

Direct delivery of antibodies in the cytoplasm of the cell was first reported in the 1980s, when it became clear that some monoclonal antibodies remained stable upon microinjection into the cell, and retained their antigen specificity [29,30,31]. For example, when embryonic fibroblasts isolated from BALB/c mice that carry an intact *Mx1* gene, which codes for an interferon inducible large GTPase with broad antiviral activity, were injected with the Mx1-specific monoclonal antibody 2C12, the cells became very susceptible to subsequent influenza A virus infection. The authors showed that these cells still expressed the Mx1 protein, yet mAb 2C12 prevented it from exerting its antiviral activity [32]. Microinjection has mostly fallen into disuse due to the high level of skill needed to perform this technique and its low-throughput nature.

### 3.2. Electroporation, an Efficient Method Associated with High Cytotoxicity

Electroporation is a very effective method for delivering proteins into a variety of cells [33]. This physical delivery technique relies on the application of an electric pulse to the cells, which results in a transient rearrangement of the cell membrane, caused by a transient reorientation of bipolar lipid molecules under an intense electric field [34]. This rearrangement results in the formation of hydrophilic pores that last from several milliseconds to seconds and allow macromolecules to cross the cell membrane into the cytosol [35,36]. When it comes to antibodies, electroporation was shown to be more efficient compared to different profection reagents, such as ProteoJuice, Bioporter, ab-DeliverIN or Pulsin [37]. However, electroporation is generally accompanied by high cytotoxicity, making it difficult to implement for certain applications [38,39]. As for any technique that includes disruption of the cell membrane, a balance must be found between efficiency and cell viability.

### 3.3. Photoporation, a High-Throughput Method with Excellent Spatial Selectivity

Direct delivery of proteins into the cell can also be achieved by laser-assisted photoporation. By focusing short (femtosecond) high-intensity laser pulses directly on the plasma membrane, transient openings are created through which macromolecules can diffuse into the cytoplasm [40,41]. More recently, it was discovered that the efficacy of this technique could be significantly increased by pre-incubating the cells with sensitizing particles, such as plasmonic gold nanoparticles (AuNPs). Lapotko and others have described that excitation of these nanoparticles with short laser pulses, led to the formation of so-called vapor nanobubbles (VNBs) [42,43,44]. These VNBs are formed when the water surrounding the nanoparticles evaporates due to heating of the AuNPs by the laser pulse. Eventually, the VNBs will seize to expand and collapse again, creating local shockwaves that transiently disrupt the cell membrane. VNB photoporation makes use of lower intensity, more diffuse laser beams, compared to the direct use of laser pulses to disrupt the cell membrane. This allows treatment of a higher number of cells, while maintaining high spatial selectivity [45]. This technique has recently been explored for the delivery of fluorescently labeled VHHs into cells, followed by high resolution imaging. Repeated photoporation of cells with fluorescently labeled VHHs allowed detailed visualization of vimentin structures in live cells [46]. Also using this technique, VHHs directed against GFP, fascin and histone H2A/H2B heterodimers were successfully used for intracellular staining in living HeLa cells [47]. Furthermore, time-lapse imaging showed that cell division and migration, as well as mitochondrial function were not affected after photoporation, indicating the value of VNB photoporation of labeled VHHs for high resolution imaging in live cells.

### 3.4. Cell Penetrating Peptide-Coupled Single Domain Antibodies Lead to Receptor Independent Cellular Uptake

The discovery that a protein can cross the cell membrane was reported simultaneously by the labs of Carl Pabo and Maurice Green, when they realized that the HIV-1 Tat (transactivator of transcription) protein could spontaneously traverse the plasma membrane of mammalian cells when added to the extracellular medium [48,49]. Soon thereafter this cell-penetrating property of the Tat protein was attributed to a 12 amino acid residue peptide (GRKKRRQRRRPQ). Moreover, it became clear that fusion of this peptide to other proteins could equally carry them across the cell membrane [50]. This elegant way of delivering proteins into cells was soon explored further and currently, about 1800 cell penetrating peptides (CPPs), which are usually 5–30 amino acid residues long and positively charged at physiological pH, have been described [51]. After the initial enthusiasm surrounding this discovery, it became clear that most proteins that are fused with a CPP remained stuck in the endosomal compartment after uptake by the cell and that the cytosolic distribution observed in earlier studies in many instances could be attributed to a fixation artefact [52,53]. Endosomal retention led the proteins to either be degraded via the lysosomal pathway or eventually recycled back to the cell membrane, meaning they never reached the cytoplasm of the cell. This feature of CPP mediated protein delivery was named the ‘endosomal escape problem’ [54]. Despite issues with endosomal entrapment, several successful examples of VHH delivery through CPPs have been reported. For example, VHHs directed against the hepatitis C virus (HCV) NS4B protein were linked to a penetratin CPP, and could reduce viral RNA levels in human hepatoma cells by 1 log value [55]. The use of arginine-rich cyclic CPPs was also demonstrated for the non-endocytic delivery of VHHs targeting proliferating cell nuclear antigen and p53 into living cells [56]. This way, VHHs were delivered directly into the cytoplasm where they were able to bind and even relocate their targets. Lastly, VHHs fused to CPPs have been used for targeting the endothelial growth factor receptor (EGFR). This receptor is overexpressed in many cancer cells, making it an attractive target for therapeutic intervention. VHHs targeting the kinase domain of the EGFR, which is localized in the cytoplasm, as well as EGFR-VHHs coupled to photosensitizers, have led to promising in vitro results in MCF-7 and A431 EGFR overexpressing cell lines, as they significantly reduced cell migration [57,58]. One of the main problems concerning the use of CPPs for therapeutic purposes is their lack of cell specificity, which results in a lower effective concentration of the therapeutic at the desired site of action and may also lead to off-target effects [59,60]. However, bio-panning of peptide-displaying phage libraries on cell types or tissues of interest has resulted in the discovery of cell type specific CPPs [61].

### 3.5. Resurfacing of Single Domain Antibodies Allows Spontaneous Crossing of Cell Membranes without Added Bulk

Polycationic resurfacing is a technique that has been used to modify the surface charge of proteins in order to increase cell permeability. To achieve this, surface exposed polar residues are replaced by positively charged arginine or lysine residues. The uptake mechanism of these positively charged proteins is most likely based on an electrostatic interaction with surface glycosaminoglycans followed by endocytosis, as was demonstrated for a supercharged +36 GFP variant [62]. Such cationization has been successfully applied for delivery of proteins and monoclonal antibodies into mammalian cells [62,63,64]. However, modification of surface charge may affect the stability and functionality of proteins. Another downside of polycationic resurfacing is the lack of standardization, meaning that the optimal amino acid substitutions need to be re-evaluated for each separate case. Therefore, in the particular case of VHHs, Bruce et al. have proposed the construction of a well-defined polycationic VHH scaffold, which can be used to graft the CDRs of VHHs that bind an intracellular target of interest [65]. Three previously reported VHHs, targeting GFP, HER2 and β lactamase, were chosen for polycationic resurfacing and subsequently examined for their structural integrity and cell penetrating properties. Determination of the VHH’s structural features using circular dichroism revealed no significant differences between the original and resurfaced VHHs, indicating that polycationic resurfacing does not lead to dramatic structural changes. GFP fusions were used to evaluate the penetration of the resurfaced VHHs into 3T3 cells. Using a flow cytometry based read-out, a concentration dependent intracellular GFP signal could be detected in the case of polycationic resurfaced VHHs, while unaltered VHHs did not enter the cells [65]. In another study, cationic VHHs that target human glial fibrillary acidic protein (GFAP), a specific marker of astrocytes, were shown to be able to cross the blood brain barrier in mice [24]. GFP fusions of these GFAP-VHHs could specifically label astrocytes in murine brain tissue sections and allowed the visualization of murine astrocytes in vivo following intracarotid or intravenous injection. Although these in vivo results are promising and provide possibilities for the use of VHHs in diagnosis and treatment of conditions of the central nervous system, cell membrane crossing VHHs still face the problem of limited tissue penetration and uneven target labeling, issues that will need to be resolved before imaging or therapeutic applications are possible.

### 3.6. Delivery by Nucleic Acids, a Straighforward Method that Is Suitable for In Vivo Delivery

Transfection of plasmids that contain VHH coding sequences is the most straightforward way to express a VHH as an intrabody. Rothbauer et al. demonstrated that GFP targeting VHHs that were expressed intracellularly following transfection of HEK293T cells, could modulate the spectral properties of their target protein [66].

Although recombinant plasmid transfection is very useful in research settings, and has been used frequently in clinical trials to elicit vaccine antigen-specific immune responses, there is some concern that injected plasmid DNA or parts thereof may insert in the genome of somatic cells. A more appropriate carrier for the transient delivery of genetic material into cells may be in vitro transcribed (IVT) RNA. Delivery via mRNA allows a transient and rapid expression of the encoded therapeutic specifically in the target organ, which allows a reduction of the administered dose and minimizes risk of toxicity. The use of mRNA encoded antibody formats has already been demonstrated in the case of extracellular targets for treatment in clinical models of viral infections, such as RSV and HIV-1, as well as for treatment of advanced tumors [67,68,69]. Zhou et al. demonstrated that transfection of in vitro transcribed nucleoside-modified RNA encoding VHHs led to efficient VHH production in A549 cells as soon as 3 h post-transfection [70]. Once expressed, the VHHs that target GFP, vimentin or histone deacetylase 6 are able to specifically bind their respective targets and remain present in the cells up to 72 h after transfection, as demonstrated by immunohistochemistry and western blotting

Another way to deliver a VHH coding sequence into cells is by viral transduction. Lentiviral VHH libraries have been used as a screening method to select for VHHs that can interfere with the replication of several viruses such as influenza A virus (IAV), vesicular stomatitis virus (VSV) and porcine reproductive and respiratory syndrome virus (PRRSV) [71,72]. When it comes to the use of VHHs for therapeutic purposes, adeno-associated viruses (AAV) are an appealing way for delivery of the VHH gene into the cell, as they rely on persistent episomal replication and expression of the encoded cargo [73]. Successful in vivo AAV delivery of an intracellular VHH was demonstrated by Li et al., who investigated the effects of a VHH that targets the phosphorylation site at the cytoplasmic side of ryanodine receptor 2 (RyR2) [74]. Chronic phosphorylation of RyR2 in cardiomyocytes leads to heart failure. Indeed, AAV-9 mediated delivery of the anti-RyR2 VHH in a rat model for ischemic heart failure not only leads to reduced phosphorylation of RyR2, but also normalized myocardium function. It is important to note however, that pre-existing immunity against AAV vectors has been identified in a large part of the population [75].

## 4. Therapeutic Potential of Intrabodies

With over 100 monoclonal antibodies currently approved by the U.S. Food and Drug Administration (FDA), mAbs are the leading therapeutic biopharmaceuticals used worldwide [76]. Still, many targets remain inaccessible to mAbs and their production costs are substantial [77]. On the contrary, expenses of VHH production can be kept low by the use of microorganism based expression systems [78]. Furthermore, VHHs can be engineered in different formats to optimize their pharmacokinetic properties [79]. In 2018, the first VHH based therapeutic, caplacizumab, was approved by the EMA for the treatment of acquired thrombotic thrombocytopenic purpura, a rare blood-clotting disease [80]. Several more clinical trials involving VHHs are currently ongoing, yet all of these therapeutics are directed at extracellular targets. Nonetheless, intracellular VHHs have also been shown to have therapeutic potential in several disease areas. In this section, we highlight reports on the use of intracellular VHHs with potential clinical applications, published from 2017 onwards. A comprehensive overview of publications concerning intrabodies with possible therapeutic applications is provided in Table 1.

### 4.1. Intrabodies with Therapeutic Potential for Cancer Therapy

When it comes to the treatment of solid tumors, VHHs may be able to outperform mAbs as their small size allows superior tumor penetration. This results in a higher effective antibody concentration and allows targeting of the entire tumor [81]. It is however important to note that a very high affinity for the antigen may also result in immobilization on the surface of the tumor and failure to penetrate, which is a challenge for both VHHs and mAbs alike. The major downside of using VHHs in cancer therapies is their fast renal clearance, resulting in half-life values of as little as half an hour. However, cell penetrable VHHs can escape this rapid clearance and thereby prolong their therapeutic activity.

The EGFR is abnormally regulated in many cancer cell types. The receptor can be overexpressed or activated without ligand interaction, leading to increased cell proliferation, migration and angiogenesis [82]. Current therapeutics targeting EGFR are either kinase inhibitors or mAbs, such as trastuzumab, that target the extracellular receptor domain, thereby blunting receptor signaling. The main issue with the use of kinase inhibitors as therapeutics remains their off-target effects, while long term treatment with mAbs may lead to selection of non-responding tumor cells [83,84]. VHHs have been explored as a new therapeutic to target the EGFR. A VHH that targets the EGFR ectodomain has been demonstrated to prevent the outgrowth of A431 derived solid tumors in a murine xenograft model by inhibiting EGFR signaling [85]. To circumvent the limited efficacy of these VHHs caused by their rapid clearing, Tabtimmai et al. isolated three VHHs, from a humanized camel phage display library, that can cross the cell membrane by linkage to a nona-arginine cell penetrating peptide. These VHHs target the cytosolic tyrosine kinase domain of the EGFR (EGFR-TK) and were approximately 1000-fold more efficient in blocking kinase activity compared to a small compound kinase inhibitor. Furthermore, all three VHHs could significantly lower cell migration of A549 cells without causing cytotoxicity [58].

Pancreatic cancer is extremely difficult to treat and marked by a highly hypoxic tumor environment. Hypoxia induced factor 1α (HIF-1α) is one of the main regulators of cell survival under hypoxic conditions and has been found to play a central role in tumor progression of pancreatic cancers, making it an interesting therapeutic target [86]. It has been demonstrated that high expression of HIF-1α can increase resistance of pancreatic tumor cells to the current first line chemotherapeutic gemcitabine and that knock down of HIF-1α combined with gemcitabine leads to significant synergistic effects [87,88]. Use of chemical HIF-1α inhibitors is associated with indirect off-target effects, while the in vivo use of CRISPR-Cas9 based delivery systems for HIF-1α knock-out remains challenging [89,90]. Hu et al. reported the isolation of a HIF-1α specific VHH via ribosome display, which was further optimized by in silico guided affinity maturation [91]. Intracellular expression of the HIF-1α VHH significantly enhanced gemcitabine cytotoxicity in pancreatic tumor cell lines, making it an interesting candidate for further therapeutic exploration. However, the main challenge for clinical application of intrabodies remains to find efficient and safe ways for their intracellular delivery.

### 4.2. Intrabodies as Therapeutic Options for Diseases Caused by Protein Misfolding

Misfolding and aggregation of α-synuclein in the brain is a hallmark of many neurodegenerative diseases. The most prominent of these conditions, Parkinson’s disease, is characterized by the presence of intracellular α-synuclein aggregates throughout the brain, called Lewy bodies. It has been suggested that neuron toxicity is mainly caused by the soluble α-synuclein oligomers, rather than the large insoluble fibrils which are formed at a later stage [92]. This makes soluble α-synuclein monomers and oligomers interesting targets for therapeutic intervention in Lewy body disorders. Guilliams et al. characterized two VHHs, derived from an immunized llama, that bind distinct epitopes in the C-terminus of α-synuclein [93]. This proved to be an interesting binding site, as the C-terminus of α-synuclein is accessible in both its soluble form, as well as in the insoluble fibrils, although binding affinity was reduced upon extensive fibril formation. Next, it was demonstrated that these VHHs, NbSyn2 and NbSyn87, both inhibit the formation of α-synuclein fibrils in vitro. Moreover, VHH binding led to a destabilization of α-synuclein fibrils and reduced cellular toxicity [94]. In order to increase the efficacy of the α-synuclein-specific VHHs, a proteasomal targeting PEST motif was added to the VHH C-terminus, which would allow the VHHs to target monomeric as well as oligomeric α-synuclein for proteasomal degradation, effectively preventing fibril formation. Indeed, fusion of NbSyn87 to a PEST motif led to rapid degradation of α-synuclein levels in vitro, along with reduced cytotoxicity [95]. In vivo proof-of-concept for the therapeutic treatment of α-synuclein aggregation induced neural damage was provided by Chatterjee et al. [96]. A viral vector encoding human α-synuclein was injected into the substantia nigra of Sprague–Dawley rats, leading to the formation of Lewy body-like inclusions. Three weeks later, animals were treated with lentiviral vectors encoding NbSyn83-PEST by stereotaxic injection into the substantia nigra. Histological analysis indicated that the treatment led to a two-fold reduction in the presence of α-synuclein aggregates compared to saline-treated control animals. Unfortunately, this reduction was also accompanied by an augmented inflammatory response, characterized by increased microglial density. Nevertheless, these studies demonstrate the potential of treating Lewy body diseases, such as Parkinson’s disease, with viral vector delivered intracellular α-synuclein targeting VHHs.

Another protein misfolding disease is gelsolin amyloidosis, an autosomal dominantly inherited affliction that leads to a plethora of neurological, ophthalmological and dermatological symptoms [97]. A point mutation in the gelsolin protein results in a partial unfolding of the second domain, revealing a cryptic furin cleavage site. Subsequent intracellular cleavage by furin followed by extracellular cleavage by MT1 matrix metalloproteinase (MT1-MMP) leads to the formation of 8 kDa and 5 kDa gelsolin plasma fragments, which are prone to aggregation. Verhelle et al. aimed at preventing the formation of these gelsolin fragments using a bispecific VHH construct that is able to protect gelsolin from both cleavage by furin and MT1-MMP [98]. Both VHHs were separated by a Gly-Ser linker and an MT1-MMP sensitive peptide. Following injection of the AAV-Nb11-FAF 1 vector in the retro orbital plexus of AGel mice, the VHH construct was detectibly expressed in the plasma up to 3-months post injection, and was associated with reduced cleavage of mutant gelsolin fragments and concomitant significantly improved muscle contractions.

The above studies underline the utility of VHHs as therapeutics in protein misfolding diseases. They can be armed with effector functions and can target specific protein domains, all while maintaining their binding abilities in the cytosol.

### 4.3. Intrabodies as Potential Therapeutics against Live-Stock Viruses

Porcine reproductive and respiratory syndrome virus (PRRSV) is an enveloped positive stranded RNA virus which causes major economic losses in the pig industry worldwide [99,100,101]. Although the emergence of this virus dates back to the 1980s, outbreaks still cannot be controlled effectively due to its rapid evolution and frequent outbreaks of highly pathogenic strains [102]. Because of its crucial role in the viral life cycle, Liu et al. have chosen PRRSV non-structural protein 4 (Nsp4) as a potential new therapeutic target [103]. Three Nsp4 binding VHHs were isolated from an immune library by phage display and their coding sequences were subsequently delivered into Marc-145 cells by lentiviral transduction. Two of these stable cell lines, producing Nb41 and Nb43 were shown to be resistant to PRRSV replication. The same research group also isolated VHHs against a second PRRSV non-structural protein, Nsp9 [104]. One particular VHH candidate, Nb9, was fused to a Tat-CPP and shown to be successfully delivered into porcine alveolar macrophages while retaining its anti-PRRSV abilities [105]. Next, a broader lentiviral-based screen was set up to identify PRRSV neutralizing VHHs [72]. In this study, the VHH repertoire from a naïve camel VHH library was delivered into Marc-145 cells, which were subsequently screened for their PRRSV resistance. This screen delivered three promising candidates, which were able to suppress PRRSV replication when expressed intracellularly. One candidate, Nb9, which the authors propose targets the viral glycoprotein, was also able to suppress PRRSV infection when delivered into Marc-145 cells via fusion with an NLS-A cell penetrating peptide. Taken together, these studies demonstrate that intracellularly expressed VHHs may be further developed as attractive new therapeutics against PRRSV.

Another virus that poses a threat to live-stock animals is bovine viral diarrhea virus (BVDV). BVDV is a positive stranded RNA virus that belongs to the *Pestivirus* genus. Infections with BVDV cause worldwide economic losses in the cattle industry and so far there is no effective treatment available [106]. Recently, the use of intracellular VHHs was also investigated as a potential therapeutic to control BDV outbreaks. Duan et al. chose the highly conserved BVDV non-structural protein 5B (NS5B), which functions as an RNA-dependent RNA-polymerase, as a target [107]. After immunization of a camel with NS5B, specific VHHs were enriched by three consecutive rounds of bio-panning. Eventually, after characterization of binding specificity and selection based on CDR3 variability, three VHHs were retained. These were introduced in MBCK cells via lentiviral transduction and tested for their ability to protect the cells from BVDV infection. One candidate, Nb1, was able to suppress BVDV infection when expressed intracellularly. The above studies demonstrate that VHHs can not only be used as therapeutics in the clinic, but can also aid in the control of infectious diseases that threaten live-stock animals.

**Table 1:** Overview of intracellular VHHs with possible therapeutic applications.

VHH Identifier	Target	Indications	Modification	Delivery	Outcome	References
**Cancer Therapies**						
R9-VH18, R9-VHH35, R9-VH36	EGFR-TK	Non-small cell lung cancer and many other cancer types	/	Nona-arginin CPP (R9)	Cell penetrable VHHs were approximately 1000-fold more efficient than conventional tyrosine kinase (TK) inhibitors in in vitro assays. R9-VH18 and R9-VH36 also significantly reduced A549 cell motility compared to TK inhibitors.	[58]
VHH212	HIF-1α	Pancreatic ductal adenocarcinoma (PDAC) tumors	/	Transient transfection	Intracellular expression of VHH212 could significantly lower the IC50 of gemcitabine in two human pancreatic cancer cell lines.	[91]
**Neurodegenerative diseases**						
VH14PEST	α -synuclein	Parkinson’s Disease	PEST proteasome targeting signal	Transient transfection in a differentiated SH-SY5Y neuronal cell line	Intracellularly expressed VH14PEST is able to significantly reduce aSyn levels and protects cells against aSyn toxicity.	[93,94,95]
VH14PEST, NbSyn87PEST	α -synuclein	Parkinson’s Disease	PEST proteasome targeting signal	AAV-5	AAV-5 mediated in vivo delivery of VH14PEST and NbSyn87PEST resulted in a 2-fold reduction of pathological aSyn aggregates.	[96]
3F5	PABPN1	Oculopharyngeal muscular dystrophy (OPMD)	NLS-GFP fusion	Transient transfection	3F5 is not only able to prevent PABPN1 aggregation, but also clears existing aggregates upon transfection in COS-1 cells	[108]
3F5, 3A9, 3E9	PABPN1	Oculopharyngeal muscular dystrophy (OPMD)	NLS fusion	*In vivo* expression in *Drosophila thoracic* muscle nuclei	In vivo expression of 3F5 prevents muscle degeneration, decreases PABPN1 mutant aggregation and restores muscle gene expression.	[109]
VHHs 1-5	Bax	Alzheimer’s disease, Parkinson’s Disease	/	Stable mammalian cell line	Intracellular expression of Bax VHHs blocks Bax function, resulting in increased resistance to apoptosis after oxidative stress.	[110]
GSN Nb11	Gelsolin	Gelsolin Amyloidosis	/	Transgenic mice expressing ER-directed GSN Nb11.	GSN Nb11 is able to shield gelsolin from aberrant proteolysis. Nb11 expressing gelsolin amyloidosis mice display improved muscle contraction.	[111]
Nb11-FAF1	Gelsolin	Gelsolin Amyloidosis	Bispecific construct separated by MT1-MMP target sequence	AAV-9	AAV-9 delivery of Nb11-FAF-1 in a gelsolin amyloidosis mouse model resulted in a significant drop in aberrant gelsolin fragments in heart and skeletal muscles.	[98]
Nb190	Rev	HIV-1	/	Transient transfection	Nb190 mimics the effects of multimerization deficient Rev mutants. Intracellular expression of Nb190 resulted in a 300-fold reduction of HIV-1 replication.	[112]
Nb190	Rev	HIV-1	/	Stable VHH expressing cell line	Nb190 expressing cells display a selective advantage upon infection and are resistant to 7 HIV-1 subtypes.	[113]
sdAb19	Nef	HIV-1	/	Retroviral transduction into fetal liver cells followed by transplantation in irradiated host	In vivo expression of sdAb19 in thymocytes led to a significant reduction of Nef induced CD4 downregulation.	[114]
BH-Int6	Integrase	HIV-1	GFP-fusion	Transient transfection	A one-step bacterial two-hybrid system allowed the isolation of of a VHH with an affinity in the low nanomolar range.	[115]
PEN-VHH6, PEN-VHH24	Non-structural protein 5B (NS5B)	HCV	/	Penetratin CPP	PEN-VHH6, PEN-VHH24 are able to significantly reduce HCV replication in vitro.	[116]
PEN-VHH7, PEN-VHH9, PEN-VH33, and PEN- VH43	Non-structural protein 4B (NS4B)	HCV	/	Penetratin CPP	NS4B VHHs are able to significantly reduce HCV replication in vitro.	[55]
VHH24, VHH28, VHH41	Serine protease	HCV	/	Penetratin CPP	VHHs are able to significantly reduce HCV replication in vitro.	[117]
PEN-VHH9	NS3 helicase	HCV	/	Penetratin CPP	Addition of penatrin coupled VHH to the culture medium significantly reduces subsequent HCV infection in vitro.	[118]
VHH S2, S3, S4, S5	S protein (HBsAg)	HBV	Fusion to ER-targeting signal and SEKDEL ER retention signal	Injection of plasmid DNA into mouse tail vein	VHH administration in a mouse model led to an increase in cellularly retained HBsAg and for some VHHs an up to 10-fold reduction of secreted HBV virions.	[119]
VHH C2, VHH C6	Core Antigen (HBcAg)	HBV	/	Transient transfection	HBcAg-specific VHHs targeted to the nucleus abrogated intracellular HBcAg signal.	[26]
Nb6	Non-structural protein 9 (Nsp9)	PRRSV	/	Stable VHH expressing cell line	Nb6 prevents the production of viral RNA and fully blocked PRRSV infection at MOI 0.01 in vitro.	[104]
Nb6	Non-structural protein 9 (Nsp9)	PRRSV	/	TAT CPP	TAT-Nb6 was efficiently delivered into porcine alveolar macrophages and efficiently inhibited the replication of several PRRSV strains.	[105]
Nb41, Nb43	Non-structural protein 4 (Nsp4)	PRRSV	/	Stable VHH expressing cell line	Intrabodies Nb41 and Nb43 protected MARC-145 cells from PRRSV induced cytopathic effect and fully blocked viral replication at an MOI of 0.001 or lower.	[103]
Nb9	Glycoprotein 5 (GP5) (Suspected)	PRRSV	/	Lentiviral transduction in MARC-15 cells or fusion to NLS-A CPP from PCV2 Cap	Lentiviral transduction of VHHs into cells proves to be an efficient method for the functional screening of PRRSV inhibiting VHHs.	[72]
Nb1	Non-structural protein 5 (NS5B)	BVDVR	/	Stable VHH expressing cell line	Nb1 is able to significantly restrict BVDVR replication at 48 h post infection in vitro.	[107]
JJX12	Ricin toxin RTA and TRB subunits	/	Bispecific construct linked by 15-mer peptide	Cellular uptake by macropinocytosis	The bispecific JJX12 binds ricin toxin on the surface of cells followed by co-internalization. JJX12 binding inhibits retrograde transport of the ricin to the TGN and traps the toxin in the late endosomes.	[120,121]
VHH5	SpvB toxin	*Salmonella typhimurium*	/	Transient transfection	When transfected into Vero cells or RAW macrophages, VHH5 is able to prevent SpvB induced actin cytoskeleton disintegration.	[122]
ALc-B8	Botulinum neurotoxin (BoNT)	*Clostridium botulinum*	YFP-fusion	Transient transfection	Transfection of Alc-B8 efficiently reduced BoNT induced SNAP25 cleavage compared to control transfected cells.	[123]
D5-B8	Botulinum neurotoxin (BoNT)	*Clostridium botulinum*	Fusion to truncated F-box domain (TrCP)	Transient transfection or lentiviral transduction	D5-B8 expressing neuronal cells recovered 2,5 times faster from BoNT intoxication than control cells.	[124]

## 5. Use of Intrabodies as Research Tools

**Figure 3:**
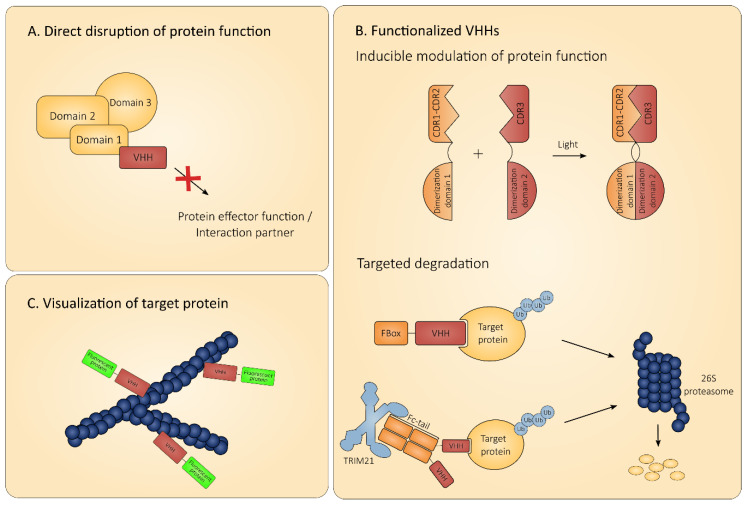
Schematic overview of the different uses of VHHs as research tools. (**A**) VHHs are able to target a particular protein domain, hereby inhibiting a specific protein effector function or a specific protein interaction. (**B**) VHHs can be coupled to functional groups. Creating a split VHH, coupled to light-sensitive dimerization domains, allows the precise timing of VHH modulation of target protein function [125]. On the other hand, coupling of VHHs to an F-box domain leads to the recruitment of cellular ubiquitin ligases, effectively marking the target protein for proteasomal degradation. Alternatively, coupling intracellular VHHs to and Fc-tail allows them to be recognized by tripartite motif-containing 21 (TRIM21), a cytosolic IgG receptor that can equally mark their target for proteasomal degradation, via its E3 ubiquitin ligase activity. (**C**) Chromobodies (CB) can be used to visualize and trace target proteins inside the cell.

Soon after the discovery of camelid heavy chain antibodies, the potential use of their variable domains as research tools was recognized. Their small size and tendency to bind cleft-like epitopes make them ideal molecules for targeting protein enzymatic sites and modulating their functionality. Moreover, due to the possibility to tag VHHs with all sorts of functional moieties, ranging from fluorophores to proteasomal degradation tags, the possibilities of intracellular VHHs as research tools are plentiful. Here, we provide an overview of recent studies in which intracellular VHHs were used to address research questions (Figure 3).

### 5.1. Intrabodies to Directly Probe Protein Functionality

An important advantage that VHHs hold against classical protein knock-down strategies, such as RNAi, is their ability to target specific protein domains, without disturbing the function of the entire protein. This can give rise to unprecedented insights in the functionality of the protein, as well as its interacting partners. In this way, it is possible to study the functionality of specific proteins and even protein domains with high precision in the context of larger protein complexes. One such example is the use of VHHs that bind distinct epitopes in the NTA domain of cortactin [126]. Cortactin is a multi-domain protein, that is involved in the activation of the Arp2/3 complex [127]. This complex is bound by the cortactin NTA domain, which leads to the formation of branched actin filaments. Meanwhile, the actin binding domains of cortactin are used to stabilize these newly formed actin branches. Actin polymerization and branching plays a role in the formation of invadopodia, which in turn are important for cancer cell extravasation and motility [128]. Two cortactin binding VHHs, Nb2 and Nb3, that bind the NTA domain of cortactin, were able to interfere with Arp2/3 mediated actin branching. It was demonstrated that, although both VHHs bind the same cortactin domain, Nb3 was able to disrupt the interaction with Arp2/3, while Nb2 was not. Additionally, it was shown that both VHHs could inhibit the formation of new actin branches in vitro, yet Nb3 expression also led to degradation of existing actin filaments. This translated to the distinct effect of the VHHs on invadopodium formation; while both VHHs interfere with the formation of new invadopodia, Nb3 also seemed to have a destabilizing effect on existing invadopodia. This study demonstrates that with intracellular VHHs, it is possible to disrupt protein function with very high resolution, without resulting in a full knock-down. These findings not only bring insight into the complex process of actin filament formation and stabilization, but can also possibly have clinical value. As the authors suggest, delineating the epitopes of the VHHs, may aid in the development of new therapeutic inhibitors of cancer cell motility.

A second example of direct interference with protein function are VHHs that interact with the Shoc2 protein, a scaffold protein that regulates and finetunes signaling through the extracellular signal-regulated kinase (ERK) 1/2 pathway [129]. Two VHHs, named B99 and B120, were isolated from a synthetic humanized VHH library, and were found to be able to bind Shoc2 with nanomolar affinity. Epitopes of the VHHs were located in a stretch of amino acids between the unstructured N-terminus of Shoc2 and the C-terminal leucine rich repeat domain. Surprisingly, it was found that intracellular expression of the VHHs led to an increase in ERK1/2 phosphorylation, which resulted in increased pathway signaling. Moreover, when VHH B99 was co-expressed with a Shoc2-S2G mutant, which impairs signaling through the ERK1/2 pathway, it could partially restore ERK1/2 phosphorylation. These results indicate that the Shoc2 epitope that is targeted by VHH B99 and B120 might be involved in a negative feedback loop that modulates ERK1/2 signaling. Binding of the VHHs to this epitope would then render it inaccessible and may result in increased signaling through the ERK1/2 pathway. In humans, the S2G mutation in Shoc2 leads to the autosomal dominant Noonan syndrome with loose anagen hair [130]. Shoc2 VHHs that are able to counteract the detrimental effects of this mutation may thus not only be useful for elucidating the mechanism of Shoc2 function, but could also be explored for their therapeutic potential.

Another example in which VHHs can very precisely interfere with protein function comes from a study published by Tome-Amat et al. [131]. Here, a previously isolated VHH targeting the nucleoprotein of IAV (NP) was used to characterize the function of NP in the import of viral ribonucleoprotein complexes (vRNPs) into the nucleus. Ashour et al. had previously demonstrated that this NP-VHH was able to interfere with IAV infection and that this restriction occurred at a step prior to nuclear import of the viral genomes [132]. It was further shown by Tome-Amat et al. that this VHH interfered with the ability of NP to bind α-importins, which is necessary for efficient nuclear import of the vRNPs. Interactions between NP and α-importins are transient and have a low affinity. The authors reasoned that in order to obtain efficient import, avidity of the interaction is likely to be important and set out to determine how many of these interactions were necessary to support nuclear vRNP import. An NP-specific VHH coupled to the NLS of the NP was used to ‘outsource’ the interaction with α-importins from NP to the VHH. In this way, by varying the ratio of NP-VHH and NP-VHH-NLS delivered to the cells, the number of accessible NLS sequences for interaction with α-importins, could be determined. From this experiment, it was estimated that about 20% of NLS sequences should be available for interaction in order to obtain efficient nuclear import. This number however varied in different cell types, and the amount of free NLS needed correlated with the infectivity of the cell line. The authors also investigated what would happen if vRNPs are retained in the cytoplasm by preventing their interaction with α-importins. It was shown that retention of vRNPs rapidly results in an upregulation of type I interferons, hereby confirming the hypothesis that IAV replication in the nucleus allows the virus to avoid cytosolic innate immune sensors. NP is a notoriously difficult target to manipulate, due to its central role in IAV infection and replication. Knock-down and genetic engineering often result in replication deficient viruses. Intracellular VHHs on the other hand, allow very precise manipulation of NP function in the limited timeframe between membrane fusion and nuclear import, again proving their value as basic research tools.

Studying protein function using VHHs offers the possibility to specifically target certain protein domains, rather than disrupting full protein function. This approach is especially useful for proteins that are difficult to manipulate, due to their essential function, or proteins that serve as scaffolds for numerous other interaction partners. The above studies nicely illustrate how the highly specific binding of VHHs can be used to pinpoint protein sites with high resolution, and how the resulting functional analysis may not only lead to new insights in protein functionality, but also shine a light on possible new therapeutic targets.

### 5.2. Functionalized Intrabodies

VHHs can be coupled to a wide range of functional groups, which allows them to modulate their targets functionality. Recent advances in the field demonstrate how functionalized VHHs can reversibly switch off the signaling activity of cellular receptors, or very precisely target certain protein conformations for proteasomal degradation. These studies highlight once more the unsurpassed precision and flexibility of using intracellular VHHs as research tools.

#### 5.2.1. Modulating Protein Function

G protein coupled receptors (GPCRs) play numerous roles in many cellular pathways. To better understand the physiological function of GPCRs, it is of interest to be able to target only a specific subset of these receptors, of which over 800 family members have been identified in the human genome [133]. In a recent study, Farrants et al. applied a novel method to reversibly switch off signaling by the G protein-coupled metabotropic glutamate receptors (mGluRs) [134]. For this purpose, the mGluR was coupled to GFP, and targeted using a well described GFP-specific VHH. This VHH was genetically fused to a SNAP-tag, a 19.4 kDa polypeptide which can readily interact with several functional groups, such as different fluorophores, dyes or photoswitches. By subsequently adding a photo switchable molecule, which interacts with the VHH-SNAP fusion, reversible activation and inactivation of the mGluR signaling was obtained, by simply switching off or on 380 nm illumination, respectively. Activity of the mGluR was monitored indirectly, by measuring the ion flow through co-transfected G protein-gated inward rectifying potassium channels, a well-defined downstream effector of mGluR, by patch clamp electrophysiology. This elegant method using VHH-photoswitch conjugates allows very precise and reversible optical control of the target protein and can easily be expanded for the study of other endogenous cellular receptors.

Another example of the use of intrabodies for the precise spacio-temporal control of target proteins was recently described by Yu et al. [125]. In this study, split intrabody fragments are coupled to blue light-inducible heterodimerization domains, creating so-called optobodies. Using a GFP binding VHH for proof of concept, it was demonstrated that in order to retain antigen binding after dimerization, the intrabody could best be split so that one part contains CDR1 and 2 and the second part holds CDR3. Next, the VHH segments were coupled to blue light-inducible dimerization domains, and it was demonstrated that dimerization could be induced with high spacio-temporal precision. The affinity of the reconstituted GFP VHH appeared to be equal to that of the original VHH. Furthermore, the GFP optobody remained intact as long as its target was present, indicating that optobody activation is irreversible due to the high affinity for its target. The authors further expanded their toolbox by developing optobodies against gelsolin and the β2 adrenergic receptor (β2AR). Upon light activation, the mito-conjugated gelsolin-specific optobody was able to translocate its target to the mitochondria, while the β2AR-specific optobody accumulated at the plasma membrane, where its target resides. Expression of the anti-gelsolin optobody in NIH3T3 cells resulted in a significant decrease in cell motility when treated with blue light, but not in the dark, as demonstrated by tracking individual cells using live cell imaging. Moreover, the motility of single cells could be manipulated by targeted blue light induction, underlining the high spatial precision of the optobody technique. Similarly, the β2AR optobody was able to inhibit β2AR signaling in HEK239T cells, by inhibition of receptor internalization. The optobody technique provides a platform for the light-inducible activation of intracellular VHHs and could be used for the manipulation of numerous endogenous targets. One case in which optobodies may also prove to be of added value is in the context of elucidating the mechanism of an antiviral protein. For example, light-inducible activation of an antiviral protein using an optobody at different time points post infection, may help to pinpoint the exact phase of the infection cycle that is targeted by this protein.

VHHs have also been used for the targeted degradation of specific protein conformational states [135]. RHOB belongs to the Ras-related family of the RHO GTPases and exists as pools of GDP-bound and GTP-bound forms in the cytoplasm [136]. RHOB distinguishes itself from other RHO GTPases by its unique C-terminus, which is prone to modifications with several post-translational modifications, which each confer a specific localization and function to RHOB. Because of this, a role for RHOB has been implied in several cellular processes, among which regulation of the DNA damage response and intracellular trafficking [137]. Despite the involvement of RHOB in many cellular processes, its molecular mechanism remains unclear. To address this, Bery et al. generated a method to selectively target GTP-bound form of RHOB for degradation, by making use of RHOB-GTP specific VHHs, which were selected from a synthetic humanized VHH library. VHHs that were able to target intracellular RHOB were selected using an elegant functional screening strategy. Enriched VHHs were cloned into a bicistronic vector that allowed the VHH to be expressed as an F-box-VHH coupled to a GFP protein with a mitochondrial targeting sequence (MitoGFP). The F-box conjugated VHH results in degradation of its target, while the MitoGFP allows for rapid detection of cells that express a functional intrabody. Transfection of these bicistronic constructs in cells that express an RFP labeled RHOB, resulted in easy detection of cells expressing a functional F-box-VHH, by selection of cells with a decreased RFP signal and green mitochondria. This selection strategy resulted in the identification of one VHH that was able to induce selective degradation of endogenous RHOB-GTP levels when expressed intracellularly. The specificity of this VHH for the GTP-bound state of RHOB, versus the GDP-bound state, was demonstrated by co-immunoprecipitation experiments. Degradation of RHOB-GTP led to significantly higher levels of DNA damage markers and a marked increase in cell migration, indicating that RHOB-GTP, but not RHOB-GDP, is responsible for maintaining genome stability and suppression of cell invasiveness. This selection method can be expanded to identify F-box-VHHs against several other family members of the small GTPases and aid in elucidating their functions.

Another recent technique for the rapid and specific degradation of endogenous proteins is based on the finding that antibody bound pathogens can be recognized by the cytosolic IgG-receptor tripartite motif-containing 21 (TRIM21) [138]. TRIM21 is a cytosolic E3 ubiquitin ligase that binds Fc-domains with very high affinity (around 0.6 nM) and rapidly recruits the proteasomal degradation machinery upon binding [138]. Inspired by these findings, Clift et al. developed a technique for the knock-down of endogenous proteins by labeling them for TRIM21-mediated degradation, an approach that they named Trim-Away [139]. The Trim-Away method is based on the delivery of recombinant TRIM21 and target-specific antibodies into cells. As a proof of concept, NIH3T3 cells that overexpress mCherry-TRIM21 and GFP were micro-injected with anti GFP antibody. This led to the rapid degradation of GFP, with a half-life of only 16 min, and was paired with a clear colocalization of the mCherry labeled TRIM21 and GFP. However, this experimental set-up was not successful for the degradation of exclusively nuclear, chromatin-associated H2B-GFP. The authors reasoned that this was likely due to the size of the antibody molecules and reasoned that much smaller VHH-Fc fusions may be able to cross the nuclear envelope. Indeed, administration of a GFP-VHH Fc-fusion construct to cells expressing H2B-GFP led to a rapid decrease of the nuclear GFP signal. The authors further demonstrated the use of Trim-Away in non-dividing primary cells, for which genome- and RNA targeting have proven to be challenging. Interestingly, the technique is also effective when only endogenous TRIM21 is present, foregoing the need of genetic engineering of the target cells or the addition of recombinant TRIM21 [139]. Combining Trim-Away with Fc-coupled VHHs may prove to be another promising technique for the rapid and precise degradation of endogenous proteins.

The above studies demonstrate how the coupling of intrabodies to functional moieties provides a plethora of possibilities to study protein functions. Other examples include using intrabodies as a tool for protein relocalization, resulting in a functional knock out [140,141,142,143], or allowing the study of protein-protein interactions [56]. More recently, Prole and Taylor have generated a toolbox of functionalized VHH intrabodies that target common fluorescent protein tags [144]. VHHs that target GFP and RFP, as well as some of their variants, were fused to many different functional modules, including fluorescent sensors for Ca^2+^ and H^+^, oligomerizing modules and SNAP-tags. This opens up many possibilities for the study of the wide range of fluorophore labeled proteins that are currently available.

#### 5.2.2. Visualization of Protein Targets

The use of fluorescent protein-conjugated VHHs, or chromobodies (CBs), is a well know application of VHH technology. The term chromobodies was first coined by Rothbauer et al., when they described a GFP binding RFP-fused VHH [66]. Chromobodies usually do not interfere with protein functionality and are able to track endogenous proteins in living cells, something that was previously impossible using classical antibodies. Moreover, the use of chromobodies abrogates the need for fluorescently labeled target proteins, which may differ significantly from their endogenous counterparts in terms of expression level, localization, stability and functionality. In this part, an overview of recent studies using chromobodies for protein visualization is given.

Chromobodies have been commonly used for the visualization and study of cytoskeleton structures. One such a component of the cytoskeleton, vimentin, has been shown to be involved in the switch to an invasive cellular phenotype in various epithelial cancer types and is thus associated with poor prognosis for cancer progression [145,146]. Intracellular expression of vimentin CBs was used to visualize the real-time dynamic changes of vimentin in A549 cells [147]. Application of this CB in vivo could aid in the further elucidation of vimentin-driven EMT processes [148]. Actin is another component of the cytoskeleton that has been extensively studied using CBs. In a recent study, Wegner et al. have used an actin CB for the in vivo visualization of neuronal actin filaments using super-resolution microscopy [149]. Furthermore, CBs have been extensively used for the study of actin filaments in several parasitic organisms, such as *Toxoplasma gondii* and *Plasmodium falciparum* [150,151,152].

Chromobodies have also been used as a tool to study RHO small GTPase activation [153]. RHO GTPases play a crucial role in the assembly of actin filaments, resulting in the formation of lamellipodia, filopodia and other actin-based structures necessary for cell migration [154]. A VHH that recognizes, but does not interfere with the activity of the active, GTP-bound forms of RHOA, RHOB and RHOC, was isolated from a synthetic library. This VHH was subsequently modified to function as a biosensor for RHO small GTPase activation, using Bioluminescence Resonance Energy Transfer (BRET). For this purpose, the VHH was N-terminally fused to a GFP^2^ acceptor molecule and co-expressed with a RenillaLuc-RHO fusion construct. Administration of the renilla luciferase substrate, coelenterazine 400a, led to a significant increase of the BRET signal in the presence of RHOA, RHOB and RHOC, indicating that the intracellular VHH can specifically interact with these three RHO isoforms. The authors further suggested that this biosensor could be used in the future for the screening of small molecule modulators of RHOA/B/C activity.

Keller et al. reported that rising of antigen levels led to a dramatic increase of the signal of stably expressed CBs [155]. This effect appeared to be common to many different CBs and was named antigen mediated CB stabilization (AMCBS). The varying CB signals corresponding to respective antigen levels, appeared to be quantitative and allowed simultaneous visualization and quantification of changing endogenous antigen concentrations. The authors set out to improve the dynamic range of this assay by screening for mutations of the N-terminal amino acid residue that destabilize the CB, thus increasing its turnover rate. This phenomenon is known as the N-end rule, which states that certain N-terminal amino acids dramatically decrease protein half-life, due to stronger interaction with E3 ubiquitin ligases [156,157]. The screening for destabilizing N-terminal amino acids was done using the ubiquitin fusion technique, which encompasses the co-translational cleavage of an N-terminal ubiquitin fusion construct [158]. In this way, a set of identical CBs could be expressed, which only differed at the site of their N-terminal amino acid. It appeared that both Phe and Arg as N-terminal amino acids resulted in a rapid degradation of the CB. These turn-over accelerated CBs were able to accurately reflect the variable levels of endogenous non-membrane associated β-catenin upon addition of specific antigen inhibitors, making it even possible to distinguish between the different kinetics of both inhibitors. With a growing number of chromobodies being engineered, the authors suggest that the time-resolved quantification of many other proteins will be possible in the near future.

Chromobodies are an excellent tool for the study of endogenous proteins in a live cell imaging context. The use of stable cell lines expressing these CBs allows straightforward tracking of proteins of interest. However, there are several downsides to the stable genomic integration of CBs, for example the increased chance of aggregation or misfolding upon expression driven by very strong promoters. Furthermore, random insertion always poses the risk of disturbing essential cellular processes. As a solution to these problems, Keller et al. have proposed a strategy to optimize the generation of stable CB cell lines [159]. Firstly, expression of the CB as N-terminal ubiquitin fusion proteins improves solubility, while also allowing insertion of an N-terminal amino acid of choice in the chromobody sequence [158]. As described above, expression of CBs with an N-terminal Arg residue results in a higher CB turnover and increased antigen sensitivity [155]. The authors also suggest the use of the elongation factor 1α (EF1α) promoter to drive CB expression, as it is less sensitive to epigenetic silencing than the commonly used CMV-promoter [160]. As a last optimization step, the CB sequence is delivered into the cells by site directed integration using the CRISPR/Cas9 technology. For this, the authors chose the adenovirus-associated virus site 1, which has been reported to be a safe harbor for genomic integration [161,162]. This approach allows the generation of stable CB producing cell lines suitable for prolonged culturing.

While fusions of VHHs to fluorescent proteins are useful for creating stable cell lines, they offer little flexibility regarding the choice of fluorophore and the addition of a bulky fluorescent protein increases the distance of the fluorophore to the target, decreasing resolution. As a solution to this problem, VHHs can also be directly coupled to fluorophores using click-chemistry. For this purpose, the VHH sequences can be engineered to contain either an extra cysteine residue [163] or an unnatural amino acid [164]. Such VHHs, directly labeled with Alexa fluor 647, have been successfully used for high-resolution imaging of nuclear pore complexes [163]. In another example, directly labeled VHHs were delivered into living cells by photoporation and used for imaging of cortactin structures [164]. This last approach also allows the visualization of short-term target perturbation, which would not be feasible using genetically encoded chromobodies.

## 6. Concluding Remarks

Many recent studies that focus on protein functionality have underlined the utility of intracellular VHHs as research tools. Intracellular VHHs can modulate their target in a myriad of ways, such as direct disruption of protein function or locking their target in a particular conformational state. Furthermore, intracellular VHHs can also be coupled to a wide range of functional groups, which allows them to be used for localization studies, sensors for enzyme activity and as proteasomal degradation tags. These studies have led to novel insights that, in the longer run, may contribute to the discovery of new therapeutic possibilities.

Therapeutic VHHs can be expressed inside cells in different ways. VHH gene delivery using viral vectors may be particularly interesting in cases of diseases caused by protein defects, such as tauopathies and other protein misfolding disorders. Other maladies, such as cancer, require the therapeutic to be delivered at the site of the lesion. Here, VHHs with intracellular targets face the problem of crossing the cell membrane, although solutions to this problem, such as fusing the VHHs to cell penetrating peptides or reshaping their surface to facilitate membrane crossing, have been demonstrated to work in vitro. Another challenge these potentially therapeutic VHHs face is efficient targeting to their site of action, while avoiding rapid clearance from circulation. A benefit of intracellular VHHs in this case, is that once therapeutic intrabodies reach their site of action and enter the cell, they are protected from renal clearance [58]. Due to their formatting flexibility, VHHs can easily be coupled to specific targeting groups, be it other VHHs or even mAbs. For example, VHHs have recently been demonstrated to be promising targeting groups for chimeric antigen receptor T cells [165]. On the other hand, coupling to the CD4-targeting mAb ibalizumab was shown to efficiently concentrate HIV-1 broadly neutralizing VHHs at the site of infection [166].

Ever since their discovery, the use of VHHs as both research tools and biopharmaceuticals has been extensively explored. The realization that VHHs retain their binding capacities in the cytoplasm of cells opened up a whole new range of proteins that could now be targeted. The use of screening methods designed for intrabody discovery and standardized frameworks that allow intracellular expression will accelerate the selection of functional intracellular VHHs and hopefully lead to many more exciting discoveries.

## 7. Boxes

### 7.1. BOX 1: Single Domain Antibody Libraries

The default procedure to obtain high affinity VHHs makes use of immune libraries. For this strategy a camelid is immunized several times with purified recombinant protein, in the presence of an adjuvant, which typically results in a very robust immune response consisting of both classical and heavy chain antibodies. After immunization, a blood sample is collected from which the peripheral blood lymphocytes are isolated. These are then used for RNA isolation followed by selective amplification of the VHH sequences by RT-PCR. The VHH sequences can then be cloned into the desired display vector [167]. Immune libraries are often the preferred source of highly specific VHHs obtained through the process of in vivo affinity maturation. In some cases, the large amount (approximately 1 mg) of purified protein needed for the immunization can pose a problem. Alternative immunization strategies however, such as DNA immunization [168,169], immunization with cells expressing the antigen [170] or immunization with inactivated viruses [71] have been proven to be successful. In cases where animal immunization is not possible, due to toxicity or lack of immunogenicity of the antigen, naïve or synthetic libraries can be used. As there is no affinity maturation in these cases, library sizes must be of a significant size (~10^9^ individual clones) and complexity in order to obtain high affinity VHH candidates [171]. Nonetheless, naïve camelid VHH libraries have been successfully used for the isolation of high affinity binders [172,173]. The first fully synthetic phage display library of humanized llama single domain antibodies (NaLi-H1) was created by Moutel et al. [174]. Starting from a highly stable VHH consensus scaffold, which was optimized for intracellular expression, synthetic variability was introduced into the CDRs based on a set of naturally occurring amino acids in these sequences. The resulting VHH library consists of 3 x10^9^ individual clones and was successfully used for the retrieval of high affinity VHHs targeting a diverse set of antigens [174]. It is important to note that some of these synthetic libraries are IP protected. Currently, some companies, such as Creative Biolabs and Hyrbigenics, offer services for design and/or screening of synthetic libraries.

### 7.2. BOX 2: Single Domain Antibody Selection Methods

The most often used method for selection of specific binders from VHH libraries is phage display [167,171]. For this technology, the amplified VHH coding sequences are cloned into a phagemid vector and transformed into *E. coli*. Infection of the bacterial cells with helper phages results in the secretion of filamentous phages that each have a VHH fused to one of their coat proteins, thus effectively combining both the VHH coding sequence and translated protein into a single organism. Phages are subsequently precipitated from the supernatant using polyethylene glycol and transferred to a micro well containing the immobilized antigen. After stringent washing to remove unbound or weakly bound phages, strong binders are eluted and used to re-infect bacterial cells. Superinfection with helper phages will again result in the production of new VHH carrying phages which have now been enriched for the target antigen. This process, called bio panning, is repeated for several rounds to obtain high affinity VHHs. VHHs can also be expressed on the surface of bacterial or yeast cells [175,176,177]. The main advantage of yeast display is the ability to screen for VHH target binding using fluorescence assisted cell sorting (FACS). Using a two-color staining procedure, VHHs can easily be selected based on both VHH expression level and affinity. Another selection method for enrichment of VHHs is ribosome display, whereby the VHH sequence is fused to an arrest sequence, which prevents the protein from being released from the ribosome during translation. This method effectively couples the VHH mRNA sequence to the newly synthesized protein, via the ribosome. These mRNA-VHH-ribosome complexes are then also used for panning on immobilized antigen. Ribosome display can be performed both in vitro and in vivo. Bencurova et al. designed a universal cassette for the synthesis of mRNA-VHH-ribosome complexes in different expression systems [178]. Addition of a myc-tag and mCherry reporter sequence allows for easy purification of ribosome complexes as well as rapid assessment of translation efficiency. For the specific enrichment of intracellular nanobodies, the use of a bacterial-two-hybrid system has been suggested [115]. Here, the antigen and the VHH library are each fused to one part of split dihydrofolate reductase. Only in case of VHH binding will the enzyme be reconstituted and can cells survive in the presence of the toxic compound trimethoprim. However, in order to obtain high affinity binding VHHs with this technique it is essential that an immune library is used as a starting point, as the in vivo affinity maturation process allows to screen a smaller number of clones.
